# Short-Term Cardiac Effects of Bariatric Surgery: Is Weight Loss Alone Sufficient in Metabolically Healthy Morbidly Obese Patients?

**DOI:** 10.3390/jcdd13060271

**Published:** 2026-06-15

**Authors:** Omer Ozkan Duman, Ummu Taş, Sedat Taş, Erkan Alpaslan

**Affiliations:** Department of Cardiology, İzmir Demokrasi University, Buca Research and Training Hospital, 35390 İzmir, Turkey; ummu.tas@idu.edu.tr (U.T.); sedat.tas@idu.edu.tr (S.T.); erkan.alpaslan@idu.edu.tr (E.A.)

**Keywords:** bariatric surgery, obesity cardiomyopathy, left ventricular hypertrophy, reverse remodeling, diastolic function

## Abstract

**Background:** Obesity is an independent and major risk factor for cardiovascular diseases. However, the presence of common comorbidities such as diabetes and hypertension makes it difficult to understand the direct impact of obesity on the myocardium. The aim of this study is to evaluate the isolated effects of weight loss achieved after bariatric surgery on left ventricular (LV) geometry and diastolic functions in individuals with the “Metabolically Healthy Obese” (MHO) phenotype. **Materials and Methods:** The study included 28 patients (Surgical Group) who underwent Laparoscopic Sleeve Gastrectomy (LSG) between January 2022 and December 2025, had a preoperative Body Mass Index (BMI) > 40 kg/m^2^, and had no known cardiovascular or metabolic diseases. The control group consisted of 25 age- and gender-matched metabolically healthy morbidly obese patients who had not undergone surgery. Demographic and echocardiographic data of all participants were analyzed at baseline and at 6 months. **Results:** Weight Loss: In the surgical group, BMI decreased from 46.21 kg/m^2^ to 37.11 kg/m^2^ at the 6th month, while no significant change was observed in the control group. Cardiac Structure: In the surgical group, Left Ventricular Mass Index was significantly decreased from 51.11 g/m^2^ to 44.57 g/m^2^. Cardiac Function: The E/A ratio, an indicator of diastolic function, increased significantly from 1.19 to 1.34 in the surgical group, indicating notable improvement. No clinically meaningful change in systolic function was detected. Metabolic Parameters: The surgical group exhibited marked improvements in glucose and lipid profiles (decrease in Total Cholesterol, increase in HDL). **Conclusions:** The study demonstrates that bariatric surgery, independent of metabolic comorbidities, directly provides “reverse remodeling” of cardiac structure and improves function through reduction of adipose tissue and alleviation of hemodynamic load. These results support the effectiveness of surgery in reducing cardiovascular risk and preserving cardiac structure even in morbidly obese patients without comorbidities.

## 1. Introduction

Obesity is a complex metabolic disorder with a prevalence that is increasing at alarming rates worldwide and is closely associated with a number of chronic diseases such as type 2 diabetes, hypertension, dyslipidemia, and coronary artery disease [1]. Obesity is considered a risk factor for cardiac dysfunction, atherosclerosis, and cardiovascular disease. When combined with other risk factors such as hypertension, dyslipidemia, and diabetes mellitus, it is referred to as “metabolic syndrome” [2]. As a global health problem with numerous medical and socioeconomic impacts, it is estimated that by 2030, obesity will affect one in every five women and one in every seven men (https://s3-eu-west-1.amazonaws.com/wof-files/World_Obesity_Atlas_2022.pdf, accessed on 8 April 2026). However, the effects of obesity on the cardiovascular system are not limited to atherosclerotic processes alone. Excess adipose tissue accumulation leads to an increase in total blood volume and consequently to elevated cardiac output. This situation increases left ventricular (LV) wall stress, which over time leads to eccentric hypertrophy, left atrial enlargement, and diastolic filling disorders; this clinical picture is referred to as “Obesity Cardiomyopathy” [3,4]. Although hemodynamic overload is a significant component in the development of obesity cardiomyopathy, it has been shown to be a multifactorial process involving neurohormonal and metabolic mechanisms [5]. Current literature suggests that weight loss can reverse cardiac remodeling and improve cardiac structure and functions [6]. Bariatric surgery is considered the most effective and long-lasting method for treating morbid obesity. There are studies in the literature showing that weight loss after surgery improves cardiac structure [7,8]. However, most of these studies have been conducted on heterogeneous groups that include diabetic and hypertensive patients. Preda and colleagues have defined obesity phenotypes as metabolically unhealthy normal weight (MUNW), metabolically healthy overweight/obese (MHO), metabolically unhealthy overweight/obese (MUO), and sarcopenic obesity (SO) [9]. It is still unknown whether the reverse cardiac remodeling that occurs after bariatric surgery depends on the magnitude of weight loss. In this context, “Metabolically Healthy Obese” (MHO) individuals provide an ideal model to isolate the pure effects of obesity on the myocardium, and no studies have been conducted on this group.

The hypothesis of this study is that weight loss achieved after bariatric surgery in metabolically healthy obese patients without comorbidities will provide reverse remodeling in cardiac structure, independent of metabolic parameters.

The aim of our study is to retrospectively examine the echocardiographic effects at the 6th month of bariatric surgery in morbidly obese patients diagnosed with MHO and to contribute to the literature by comparing the findings with a control group that did not undergo surgery.

## 2. Methods

### 2.1. Study Design

This study was conducted as a retrospective cohort study in strict adherence to the principles outlined in the declaration of Helsinki (revised in 2013). Prior to any data extraction, formal approval for this study was obtained from the Institutional Review Board (Ethics Committee) of İzmir Demokrasi University Buca Seyfi Demirsoy Training and Research Hospital (Approval Date: 28 January 2026, Approval Number: 2026/01-04). This ethical approval explicitly permitted the retrospective review and analysis of pre-existing medical records of patients who had received standard-of-care clinical evaluations and underwent Laparoscopic Sleeve Gastrectomy (LSG) between January 2022 and December 2025. All clinical procedures had been previously performed in accordance with the ethical standards of the institutional and national research committee and with the 1964 Helsinki Declaration and its later amendments.

### 2.2. Sample Selection

The study group (Surgical Group) was selected from patients with a preoperative Body Mass Index (BMI) > 40 kg/m^2^. MHO status was confirmed using NCEP-ATP III criteria: patients were required to have a fasting glucose <100 mg/dL, triglycerides <150 mg/dL, HDL ≥40 mg/dL (men) or ≥50 mg/dL (women), blood pressure <130/85 mmHg [10]. On the other hand, the patients diagnosed with Type 2 Diabetes (DM), Hypertension (HT), coronary artery disease (CAD), valvular disease, or thyroid dysfunction were excluded from the study. The control group (*n* = 25) was identified retrospectively by consecutively screening outpatient clinic records during the same study period to find healthy morbidly obese individuals who met the same MHO criteria and possessed complete baseline and 6-month follow-up echocardiographic data. These patients were then matched to the surgical group in terms of age and gender. They had not undergone surgery but were prescribed a standard diet and lifestyle program according to current guidelines [11,12]. Due to the retrospective nature of the study, strict quantitative monitoring of adherence to lifestyle interventions was not feasible; adherence was assessed solely based on patient self-reports during routine clinical follow-ups.

### 2.3. Echocardiographic Evaluation

Two-dimensional (2D) and Doppler echocardiographic recordings of each patient were performed in the left lateral position by the same operator using an ultrasound system (Vivid S60N, GE Systems, Oslo, Norway) with a 2.5 MHz transducer, in accordance with the guidelines of the American Society of Echocardiography and the European Association of Cardiovascular Imaging [13]. Measurements of the ascending aorta (Asc.Ao), interventricular septum (IVS), left ventricular posterior wall (LVPW), and left atrium (LA) were taken at end-diastole in the parasternal long axis. Left ventricular ejection fraction (LVEF), left ventricular end-systolic volume (LVESV), and left ventricular end-diastolic volume (LVEDV) were calculated using the modified Simpson’s rule from the apical four-chamber view. Early (E) and late (A) diastolic peak flow velocities were measured at the tips of the mitral valve using Pulsed-Wave (PW) Doppler, and the E/A ratio was calculated.

Left Ventricular Mass Index (LVMI) was calculated offline using the Devereux formula for left ventricular myocardial mass, based on echocardiographic measurements with the following equations, and indexed (g/m^2^) according to Body Surface Area (BSA) calculated by the Mosteller formula [14,15].

LVmass (g): 0.8 (1.04 ([LVIDD + PWTD + IVSTD]^3^ − [LVIDD]^3^)) + 0.6 g (Devereux)

BSA (m^2^) = √ height (cm) × weight (kg)/3600

LVmass index (g/m^2^): 0.8 (1.04 ([LVIDD + PWTD + IVSTD]^3^ − [LVIDD]^3^)) + 0.6 g/BSA

While the primary endpoints of this study were the changes in left ventricular geometry (LVMI) and diastolic function (E/A ratio), the evaluations regarding the ascending aortic diameter and the presence of mitral regurgitation were predefined as exploratory endpoints.

### 2.4. Statistical Analysis

In this study, certain parameters were calculated to test the preoperative and postoperative values of 53 participants (25, control group; 28, surgical group) along with their demographic information. The data obtained were analyzed using the IBM SPSS (Statistical Package for the Social Sciences) 26.0 software package. During the analysis process, descriptive statistics were first examined. Given the sample sizes of the groups (*n* < 50), the Shapiro–Wilk test was conducted to assess the normal distribution of the continuous variables. Because the primary continuous variables successfully met the normality assumption (*p* > 0.05), parametric methods (Independent Samples *t*-test and Paired Samples *t*-test) were applied. In order to identify the differences in the 0–6-month values of the control and surgical groups, Independent Samples *t*-test and Paired Samples *t*-test were applied. In all analyses, the statistical significance (*p*) value was set at 0.05. In analytical findings where the *p*-value was less than 0.05, statistical significance was considered present; otherwise, it was considered not statistically significant. Given the lack of specific literature regarding the precise magnitude of reverse cardiac remodeling in the purely Metabolically Healthy Obese (MHO) phenotype, the effect size for power calculation was estimated based on the moderate-to-large structural improvements (e.g., LVMI reduction) observed in broader bariatric surgery cohorts [16]. Assuming an effect size of d = 0.75, a Type I error rate of α = 0.05, and a power of 0.80, the minimum required sample size was calculated as 46 (at least 23 subjects per group). Our final sample size of 53 was therefore adequately powered for the primary outcomes. Additionally, to address potential allocation bias inherent in the retrospective design and to strengthen causal inference, a multivariable adjustment (ANCOVA) was performed. The effect of the surgical intervention on structural (LVMI) and functional (E/A ratio) cardiac outcomes at 6 months was evaluated by adjusting for baseline confounding factors, including age, gender, and baseline BMI.

## 3. Results

The comparison of the demographic and clinical data of the surgical group (*n* = 28) and the control group (*n* = 25) included in the study is presented in Table 1. No statistically significant difference was found between the surgical group (*n* = 28) and the control group (*n* = 25) included in the study in terms of baseline demographic characteristics and clinical parameters. The age and gender distributions of the groups were similar; likewise, there was similarity between the groups regarding smoking (Surgical: 32.1%; Control: 24.0%).

### 3.1. Demographic and Clinical Findings

**Figure 1:**
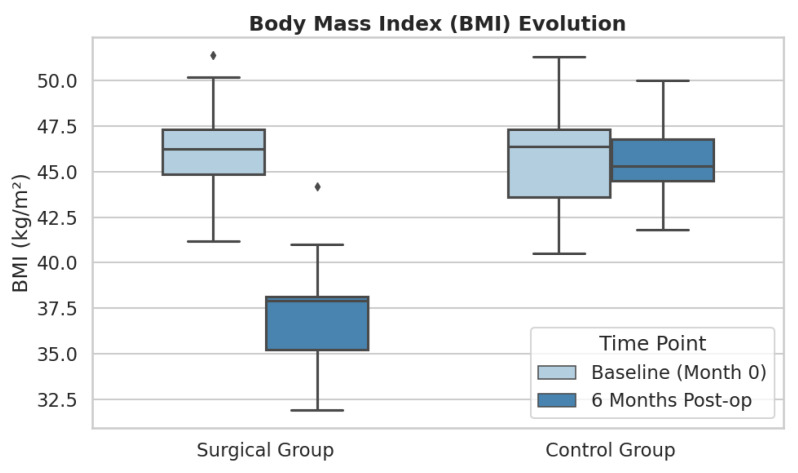
Comparison of Body Mass Index (BMI) Evolution between Surgical and Control Groups. This figure illustrates the significant reduction in BMI within the Surgical Group from Baseline (Month 0) to 6 Months Post-op (*p* < 0.001). In contrast, the Control Group showed no statistically significant change in BMI over the same period. This highlights the rapid and effective weight loss achieved through bariatric surgery. The diamond symbols indicate statistical outliers.

The initial Body Mass Index (BMI) values were measured as 46.21 kg/m^2^ in the surgical group and 45.99 kg/m^2^ in the control group, with no significant difference observed between the groups (*p* = 0.760). However, by the end of the 6th month, the BMI value in the surgical group dropped to 37.11 kg/m^2^, while it remained at 45.60 kg/m^2^ in the control group; this change is statistically highly significant (Figure 1, *p* < 0.001). Similarly, although heart rate, systolic blood pressure (SBP), and diastolic blood pressure (DBP) values were similar at the beginning, significant decreases in favor of the surgical group were recorded at the 6th month (*p* < 0.001).

### 3.2. Laboratory Parameters

In an examination of glycemic control and lipid profile, no significant difference was found between the groups in terms of baseline values (*p* > 0.05), while a marked improvement was observed in the surgical group in the sixth-month data.

Fasting glucose levels in the surgical group decreased to 85.93 mg/dL, and HbA1c values dropped to 5.65% (*p* < 0.001). No improvement was observed in these values in the control group; in fact, there was a slight increase in HbA1c (5.89%). Total cholesterol values in the surgical group decreased significantly to 171.25 mg/dL (*p* < 0.001). A significant increase was observed in HDL cholesterol levels in the surgical group (45.46 mg/dL); meanwhile, in the control group, this value (40.24 mg/dL) remained almost unchanged compared to baseline (*p* > 0.05). In light of these findings, it can be said that surgical intervention has a statistically very strong beneficial effect on weight loss, blood pressure, glycemic control, and lipid profile at the end of the sixth month.

### 3.3. Echocardiographic Parameters

The baseline and 6th-month echocardiographic parameters of the study groups are presented in Table 2. At the 6th month following surgical intervention, statistically significant and marked improvements were observed in the structural parameters of the left ventricle.

**Figure 2:**
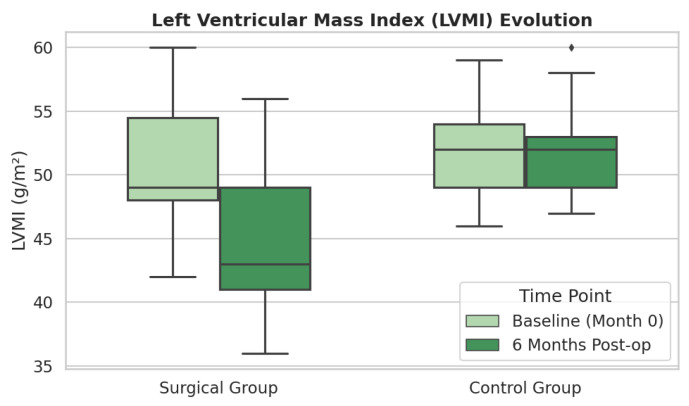
Impact of Weight Loss on Left Ventricular Mass Index (LVMI). The boxplot shows a substantial decrease in LVMI (g/m^2^) in the Surgical Group 6 months after surgery, indicating a positive “reverse cardiac remodeling” effect. No significant change in cardiac mass was observed in the Control Group. The diamond symbols indicate statistical outliers.

**Figure 3:**
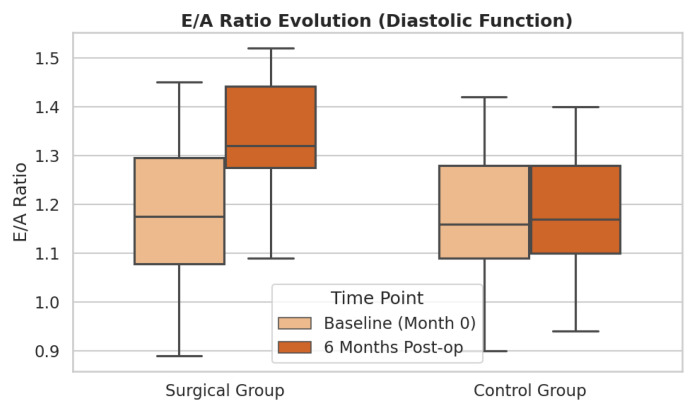
Improvement in Diastolic Function (E/A Ratio). The E/A ratio, a marker of left ventricular diastolic function, showed a significant increase in the Surgical Group at the 6-month follow-up (*p* < 0.001). This suggests that weight loss and metabolic changes after surgery lead to improved cardiac relaxation and filling.

The Surgical Group experienced a significant reduction in LVMI value of 51.11 g/m^2^ decreased to 44.57 g/m^2^ at the 6th month compared to the Control Group (Figure 2, *p* < 0.001). Furthermore, the interventricular septum (IVS) and left ventricular posterior wall thickness (LVPWd) significantly decreased in the surgical group (*p* < 0.001). IVS values decreased from 10.43 mm to 9.64 mm, and LVPWd values decreased from 10.11 mm to 9.32 mm. Left ventricular end-diastolic volume (LVEDV) and end-systolic volume (LVESV) significantly decreased in the surgical group (*p* < 0.001), while they remained stable in the control group. Left ventricular ejection fraction (LVEF) increased statistically significantly from 60.71% to 61.32% in patients who underwent surgery (*p* = 0.027). No significant difference was detected in LVEF values in the control group (*p* = 0.739). The E/A ratio, an important indicator of diastolic function, showed a significant improvement in the surgical group, rising from 1.19 to 1.34 (*p* < 0.001). In the control group, there was no change between the baseline and 6th month values (Figure 3). The left atrium (LA) diameter showed a slight increase in the surgical group, rising from 35.75 mm to 36.11 mm (*p* = 0.005). Similarly, in the surgical group, there was a statistically significant but clinically limited increase in the ascending aorta (Asc. Aorta) diameter (from 31.79 mm to 32.25 mm, *p* = 0.003). No significant change was observed in these parameters in the control group.

**Figure 4:**
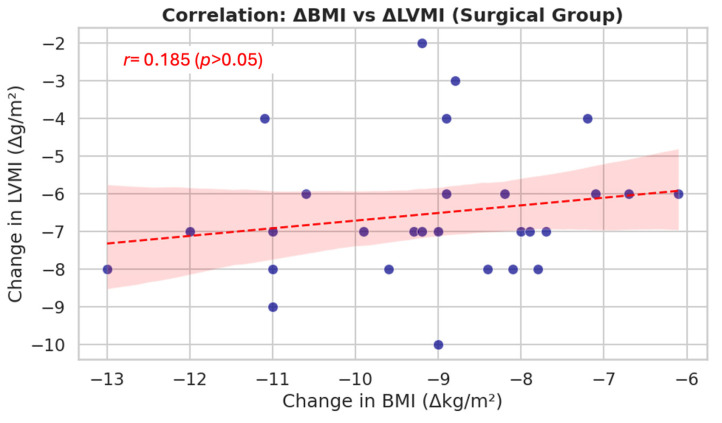
Correlation between BMI Reduction (ΔBMI) and LVMI Reduction (ΔLVMI) in the Surgical Group. The scatter plot examines the relationship between the magnitude of weight loss and the degree of cardiac mass reduction. A weak positive correlation (r = 0.185, *p* > 0.05) was found, suggesting that while weight loss is a key factor, other metabolic or hormonal mechanisms may also play a role in the observed cardiac improvements. The red dashed line represents the linear regression line, and the red shaded area indicates the 95% confidence interval.

Although both BMI and LVMI decreased significantly, correlation analysis revealed a weak positive relationship (r = 0.185) between the degree of BMI reduction and LVMI reduction in the Surgical Group (Figure 4). In the multivariable adjustment model controlling for baseline age, gender, and preoperative BMI, surgical intervention remained a significant independent predictor for both the reduction in LVMI (adjusted *p* < 0.05) and the improvement in the E/A ratio (adjusted *p* < 0.05).

## 4. Discussion

This study aimed to examine the short-term effects of weight loss achieved after laparoscopic sleeve gastrectomy (LSG) on cardiac structure and function in metabolically healthy morbidly obese (MHO) patients. The main finding of our study is that, even in patients without a known cardiovascular comorbidity, there was a significant reduction in the left ventricular mass index (LVMI) and a notable improvement in diastolic function (E/A ratio) at the 6th month following surgery. These results support that “reverse remodeling” of the heart is possible with weight loss in the early stages of obesity cardiomyopathy.

Obesity is hemodynamically considered a “high output” condition and increases the heart’s preload. As a result, it has been shown to frequently cause concentric left ventricular hypertrophy in men, and both concentric and eccentric hypertrophy in women [17,18]. In our study, the significant decrease observed in LVMI can be explained by the reduction in mechanical load. In the current literature, a comprehensive meta-analysis conducted by N. Sargsyan et al. [16] in 2024 found that bariatric surgery leads to an average 12% reduction in left ventricular mass index, which supports our study’s findings. When evaluating functional parameters, the earliest finding of cardiac involvement due to obesity is generally diastolic dysfunction. In our study, the E/A ratio increased significantly after surgery (from 1.19 to 1.34). Although we recognize that the E/A ratio alone is a basic parameter and lacks the comprehensiveness of advanced tissue Doppler imaging (such as the E/e’ ratio or LAVI) for formal diastolic grading, its significant improvement in our cohort still provides an early, detectable signal of enhanced ventricular relaxation. This finding is consistent with previous research indicating that weight loss improves fundamental diastolic filling dynamics [19,20]. Regarding systolic function, LVEF values were within normal range at baseline in both groups (60.71% and 61.16%, respectively) and did not change meaningfully after surgery. Since the initial LVEF values were already within normal limits, it is expected that changes in systolic functions would not be as dramatic as those in diastolic parameters and should be interpreted as a null finding for systolic function: bariatric surgery did not impair systolic performance, but neither did it produce clinically meaningful systolic improvement in this MHO cohort with preserved baseline ejection fractions. In our study, in parallel with the decrease in BMI provided in the surgical group (from 45.60 to 37.11), statistically highly significant reductions were observed in the thickness of the left ventricular posterior wall (LVPWd) and interventricular septum (IVS). Although surgical intervention led to highly significant reductions in both BMI and LVMI at the group level, individual correlation analysis revealed only a weak and statistically non-significant relationship (r = 0.185, *p* > 0.05) between the magnitude of BMI reduction (ΔBMI) and LVMI reduction (ΔLVMI). This is a critical finding that challenges the assumption of a simple, linear ‘dose–response’ relationship between total body weight loss and cardiac reverse remodeling. The weak correlation suggests that reverse cardiac remodeling is not strictly dictated by the absolute reduction of global adiposity (predominantly subcutaneous fat, which heavily influences BMI).

Instead, we hypothesize that the structural improvement is mediated by more complex, rapid physiological shifts independent of total mass. First, bariatric surgery induces immediate hemodynamic unloading, including reductions in total blood volume, sympathetic nervous system overactivity, and ventricular wall stress, which can precede massive weight loss. Second, as elegantly highlighted in recent multimodal imaging literature, specific visceral fat depots—particularly epicardial adipose tissue (EAT)—play a more direct and aggressive role in myocardial remodeling through local paracrine and inflammatory mechanisms than general subcutaneous adiposity [21]. The lack of a strong correlation in our findings implies that rapid hemodynamic stabilization and the targeted regression of EAT might be the true critical drivers of reverse remodeling, rather than the simple numerical drop in BMI. Additionally, the significant decrease in systolic and diastolic blood pressures suggests that ventricular load has decreased, which, in turn, appears to contribute to structural regression.

One of the most original aspects of our study is the findings obtained in patients with the “Metabolically Healthy Obese” (MHO) phenotype. In the literature, most bariatric surgery studies have been conducted with heterogeneous groups that include diabetic and hypertensive patients. However, our study demonstrates that, independent of the additional burden caused by metabolic disorders, even the reduction of adipose tissue alone can improve myocardial structure. Improvements in fasting glucose, HbA1c, and lipid profiles in the surgical group reveal that, even though MHO individuals are initially considered “healthy,” they benefit metabolically and cardiologically from weight loss.

In our exploratory analysis regarding vascular changes, although a significant regression was observed in the structure of the left ventricle, a statistically significant but clinically limited increase (from 31.79 mm to 32.25 mm) in the diameter of the ascending aorta was detected in the surgical group. While it has been reported in the literature that weight loss after bariatric surgery generally leads to a reduction in aortic diameters, it is suggested that longer follow-up periods may be necessary for this effect to become evident [22]. The possible reason for the minimal increase and the preservation of the aortic diameter observed in this study may be that vascular remodeling progresses more slowly than the change in left ventricular mass [23]. The reorganization of the extracellular matrix and elastic fibers in the aortic wall is a process that takes time, and the 6-month follow-up period may have been insufficient for the expected “anatomical regression” in aortic diameter to manifest. Additionally, it may be related to a shift in the balance between the increase in the elastic properties (distensibility) of the aorta and the changes in blood pressure following weight loss [24].

Another noteworthy exploratory finding of our study is that, although the majority of patients in the surgical group showed improvement in cardiac function, mild mitral regurgitation (MR) that was newly developed or persistent was detected in 5 patients at the 6th postoperative month. In the literature, weight loss is generally known to improve functional mitral regurgitation by reducing ventricular load. However, rapid weight loss and the ensuing rapid “reverse remodeling” process can lead to a temporary mismatch between ventricular geometry and valve structure. This situation can be explained by the “Leaflet-Annulus Mismatch” theory: while the left ventricular cavity volume and mitral annular diameter shrink rapidly, the surface area of the mitral valve leaflets remains the same. Within the smaller ventricular geometry, the relatively “redundant” leaflets may not achieve complete coaptation, resulting in regurgitation [25,26,27]. Although this condition observed in our study did not result in clinical symptoms, it was evaluated as an adaptive response to the rapid changes in cardiac geometry.

There are some limitations to our study. First, the study has a retrospective, non-randomized design and includes single-center data. The absence of randomization introduces an inherent allocation bias; it is possible that patients who elected to undergo surgery systematically differed from the control group in terms of unmeasured confounders such as intrinsic motivation, lifestyle choices, or dietary adherence. Second, due to the strict exclusion criteria applied to meet the MHO criteria, the sample size is relatively small. While our power calculation indicates adequate statistical power for the primary endpoints (LVMI and E/A ratio), we acknowledge that the study is underpowered to draw definitive conclusions regarding exploratory secondary endpoints (such as aortic remodeling or mitral regurgitation) or to perform comprehensive subgroup analyses. Third, although the 6-month follow-up period is sufficient to demonstrate early effects, it is insufficient to evaluate long-term vascular remodeling processes such as aortic and LA diameter normalization. Longer-term studies are needed to evaluate long-term processes such as aortic remodeling. However, the selection of a “pure” MHO patient group without comorbidities is the most important factor that increases the reliability of the results and eliminates confusion in the literature. Fourth, due to the retrospective nature of this study relying on standard-of-care preoperative clinical echocardiograms, diastolic function assessment relied solely on the E/A ratio. A formal grading of diastolic dysfunction incorporating tissue Doppler-derived E/e’ ratio, tricuspid regurgitation velocity, and LA volume index (LAVI) per current ASE/EACVI 2016 guidelines [28]. could not be performed due to incomplete archived data. Furthermore, the absence of speckle-tracking echocardiography (Global Longitudinal Strain, GLS) limits our ability to evaluate subclinical systolic dysfunction, which might be present despite the preserved left ventricular ejection fraction observed in our cohort. Future prospective studies should incorporate these advanced echocardiographic modalities for a more comprehensive assessment. Fifth, due to the retrospective nature of the study, we were unable to systematically quantify epicardial adipose tissue (EAT) thickness. Given its direct paracrine and inflammatory influence on the myocardium, future prospective studies utilizing comprehensive multimodal imaging or dedicated echocardiographic EAT measurements are warranted to better elucidate the mechanisms of reverse remodeling. Despite these limitations, the selection of a rigorously defined MHO cohort without comorbidities represents a meaningful methodological strength that isolates the direct hemodynamic effects of weight loss on cardiac remodeling.

In conclusion, this study has demonstrated that bariatric surgery leads to improvements in cardiac structure and function independently of metabolic comorbidities, directly through the reduction of adipose tissue and alleviation of hemodynamic load. These findings support that bariatric surgery is an effective treatment option for reducing cardiovascular risk and preserving cardiac structure in morbidly obese patients, even in the absence of comorbidities.

## Figures and Tables

**Table 1:** Comparison of the Demographic and Clinical Data of the Study Groups.

Variables	Surgical Group (*n* = 28)	Control Group (*n* = 25)	*p*-Value
Age (Year)	43.17	42.96	ns
Gender			
Female, *n* (%)	23 (82.1%)	21 (84.0%)	ns
Male, *n* (%)	5 (17.9%)	4 (16.0%)	ns
Body Mass Index (kg/m^2^)			
Baseline	46.21	45.99	0.760
6th Month	37.11	45.60	<0.001
Smoking, *n* (%)			
No	19 (67.9%)	19 (76.0%)	ns
Yes	9 (32.1%)	6 (24.0%)	ns
Heart Rate (beats/min)			
Baseline	92.89	93.60	ns
6th Month	84.54	92.32	<0.001
Systolic Blood Pressure (mmHg)			
Baseline	131.11	130.20	0.345
6th Month	124.25	129.36	<0.001
Diastolic Blood Pressure (mmHg)			
Baseline	84.14	83.68	0.593
6th Month	79.18	82.72	<0.001
Fasting Glucose (mg/dL)			
Baseline	92.07	91.88	0.737
6th Month	85.93	92.52	<0.001
HbA1c (%)			
Baseline	5.80	5.83	0.263
6th Month	5.65	5.89	<0.001
Total Cholesterol (mg/dL)			
Baseline	188.32	186.36	0.390
6th Month	171.25	185.08	<0.001
HDL Cholesterol (mg/dL)			
Baseline	40.18	39.92	0.735
6th Month	45.46	40.24	<0.001

ns: Not significant.

**Table 2:** Comparison of Echocardiographic Parameters and Effect Sizes (Cohen’s d).

	Surgical Group				Control Group			
Parameters	Baseline	6th Month	*p*-Value	Cohen’s d	Baseline	6th Month	*p*-Value	Cohen’s d
LVEF (%)	60.71 ± 1.96	61.32 ± 1.68	0.027	0.443	61.16 ± 1.68	61.24 ± 1.56	0.739	0.067
LVMI (g/m^2^)	51.11 ± 5.19	44.57 ± 4.87	<0.001	3.642	52.12 ± 3.72	52.24 ± 3.49	0.450	0.154
LVEDV (mL)	97.57 ± 18.7	94.89 ± 17.9	<0.001	0.873	97.92 ± 17.07	98.16 ± 17.2	0.491	0.140
LVESV (mL)	41.18 ± 5.89	39.11 ± 5.63	<0.001	1.440	40.96 ± 6.29	41.08 ± 5.96	0.671	0.086
IVS (mm)	10.43 ± 0.96	9.64 ± 0.73	<0.001	1.576	10.60 ± 0.87	10.72 ± 0.79	0.327	0.200
LVPWd (mm)	10.11 ± 0.69	9.32 ± 0.61	<0.001	1.576	10.08 ± 0.70	10.12 ± 0.67	0.574	0.114
Asc. Aorta (mm)	31.79 ± 1.37	32.25 ± 1.14	0.003	0.624	31.96 ± 1.57	32.08 ± 1.41	0.185	0.273
LA (mm)	35.75 ± 1.38	36.11 ± 0.99	0.005	0.575	35.96 ± 1.31	36.04 ± 1.17	0.538	0.125
E/A Ratio	1.19 ± 0.16	1.34 ± 0.12	<0.001	2.899	1.19 ± 0.13	1.19 ± 0.12	0.862	0.035

LVEF: Left Ventricular Ejection Fraction; LVMI: Left Ventricular Mass Index; LVEDV: Left Ventricular End-Diastolic Volume; LVESV: Left Ventricular End-Systolic Volume; IVS: Interventricular Septal Thickness; LVPWd: Left Ventricular Posterior Wall Diameter; Asc. Aorta: Ascending Aorta Diameter; LA: Left Atrium Diameter; E/A Ratio: Early-to-Late Diastolic Transmitral Flow Velocity Ratio.

## Data Availability

The data presented in this study are available on request from the corresponding author. The data are not publicly available due to privacy and ethical restrictions.
